# Ultrasound Doppler Flow Parameters Are Independently Associated with Renal Cortex Contrast-Enhanced Multidetector Computed Tomography Perfusion and Kidney Function

**DOI:** 10.3390/jcm12062111

**Published:** 2023-03-08

**Authors:** Arkadiusz Lubas, Arkadiusz Zegadło, Emilia Frankowska, Jakub Klimkiewicz, Ewelina Jędrych, Stanisław Niemczyk

**Affiliations:** 1 Department of Internal Diseases Nephrology and Dialysis, Military Institute of Medicine—National Research Institute, 04-141 Warsaw, Poland; 2 Department of Radiology, Military Institute of Medicine—National Research Institute, 04-141 Warsaw, Poland; 3 Department of Anesthesiology and Intensive Care, Military Institute of Medicine—National Research Institute, 04-141 Warsaw, Poland

**Keywords:** kidney perfusion, resistive index, Doppler, computer tomography, blood flow

## Abstract

Background: The assessment of kidney perfusion has an emerging significance in many diagnostic applications. However, whether and which of the ultrasound Doppler parameters better express renal cortical perfusion (RCP) was not shown. The study aimed to prove the usefulness of Doppler ultrasound parameters in the assessment of RCP regarding low-dose contrast-enhanced multidetector computer tomography (CE-MDCT) blood flow. Methods: Thirty non-stenotic kidneys in twenty-five hypertensive patients (age 58.9 ± 19.0) with mild-to-severe renal dysfunction were included in the study. Resistive index (RI) and end-diastolic velocity (EDV) in segmental arteries, color Doppler dynamic RCP intensity (dRCP), RI (dRI), pulsatility index (dPI), and CE-MDCT blood flow (CBF) in the renal cortex were estimated. Results: CBF correlated significantly with age, estimated glomerular filtration rate (eGFR), RI, EDV, dRI, dPI, and dRCP. In separate multivariable backward regression analyses, RI (R^2^ = 0.290, *p* = 0.003) and dRCP (R^2^ = 0.320, *p* = 0.001) were independently associated with CBF. However, in the common ultrasound model, only dRCP was independently related to CBF (R^2^ = 0.317, *p* = 0.001). Only CBF and EDV were independently associated with eGFR (R^2^ = 0.510, *p* < 0.001). Conclusions: Renal cortical perfusion intensity is the best ultrasound marker expressing renal cortical perfusion. In patients with hypertension and kidney dysfunction, renal resistive index and end-diastolic velocity express renal cortical perfusion and kidney function, respectively.

## 1. Introduction

The wide accessibility of radiologic imaging methods enabled the assessment of renal perfusion, which has an emerging significance in many diagnostic applications. As organ perfusion is considered a prerequisite of its function, alterations in renal perfusion can be detected, among others, in hypertension, cardiac and thyroid abnormalities, renal artery stenosis, and acute and chronic kidney diseases [1,2,3,4,5].

Most quantitative perfusion assessment methods, such as scintigraphy, computed tomography (CT), magnetic resonance imaging, and contrast-enhanced ultrasonography (CEUS), require a contrast agent for adequate measurement. However, in conventional (non-contrast) ultrasonography, some ultrasound Doppler parameters, e.g., flow velocities and resistive and pulsatility indexes (RI, PI) describing properties of intravascular blood flow, are often recognized as renal perfusion markers. It was not univocally shown which of these parameters, in a better manner, expresses the amount of organ perfusion. On the other hand, some studies challenge significant relations of considered Doppler parameters with perfusion attributes and arrange them as vascular and systemic hemodynamic properties markers [6,7,8,9]. Recently, Abe et al., in a group of 162 patients with chronic kidney disease, showed an independent association between interlobar end-diastolic velocity (EDV) and renal function, but there was no such association with regard to RI [10].

As tissue perfusion is defined as volume flow in time unit through tissue mass, e.g., mL/100 g/min, and can be estimated as blood flow in contrast-enhanced multidetector computer tomography (CE-MDCT), two-dimensional Doppler ultrasound can measure flow parameters through a plain vessel section. Probably, discrepancies resulted from the lack of third dimension between computer tomography and conventional ultrasound examination, and different methods and algorithms used in flow calculation influence measurement similarity. Lastly, a new experimental three-dimensional ultrasound perfusion assessment method was introduced, which could overcome the abovementioned inconsistencies in results [11].

Recently, many scientific publications have used the renal resistive index as an easily accessible marker of renal perfusion, especially in critically ill patients [9,12,13,14]. However, this should be confirmed in relation to an independent imaging method. Moreover, other ultrasound Doppler parameters, especially achieved in novel measurement methods, should be considered valuable in assessing renal perfusion.

The study aimed to analyze the usefulness of selected Doppler ultrasound parameters in estimating renal cortex perfusion in comparison to low-dose CE-MDCT renal cortical blood flow.

## 2. Materials and Methods

Data from non-stenotic kidneys in hypertensive patients with mild-to-severe renal dysfunction examined for renal artery stenosis were included in the study. Patients underwent ultrasound Doppler examination and then were diagnosed in low-dose CE-MDCT. Inclusion criteria encompass age over 18 years, suspicion of renal artery stenosis based on anamnesis or results of other examinations, and written informed consent. Regarding medical history and actual results, the exclusion criteria comprised acute kidney injury, chronic kidney disease (CKD) stage G5, inflammation, and iodide contrast intolerance. Informed consent was obtained from all patients who attended the study.

### 2.1. Renal Function Assessment

A morning blood sample was taken before CE-MDCT to assess serum creatinine and calculate the estimated glomerular filtration rate (eGFR) according to the CKD-EPI equation [15].

### 2.2. Contrast-Enhanced Multidetector Computer Tomography

The perfusion assessment was performed in the dynamic measurement of blood flow with a contrast agent in the three-dimensional region of interest (ROI) appointed in the renal cortex. In the time of an iso-osmolar contrast agent (Visipaque 320), intravenous infusion ROI was repeatedly scanned (single-source DECT scanner with rapid kVp switching Discovery CT 750 HF; GE Healthcare, Waukesha, WI, USA). Flow readings acquired in ROI were normalized to those obtained in the aorta. Thus, results were not dependent on contrast agent concentration. At the time of examination, patients were asked for slow and shallow breathing to avoid artifacts.

#### 2.2.1. CE-MDCT Protocol

Radiographic examination comprised two phases. The first phase was performed for localizing kidneys and included a native, helical scan of the abdominal cavity, starting from diaphragm domes up to aortic bifurcation. In the second phase, the length of the scan area was set to 14 cm to complete both kidneys’ coverage. Every tested individual received 25 shuttle passes, providing 375 images. The total acquisition time of the second phase was 42.6 s. During the examination, a nonionic contrast medium (320 mg/mL Visipaque, GE Healthcare) was administered to each patient at a rate of 4.0 mL/s using a power injector. The scan delay was set AT 10 s. Technical data of the protocol are shown in Table 1.

#### 2.2.2. Quantitative Analysis of Perfusion

**Figure 1:**
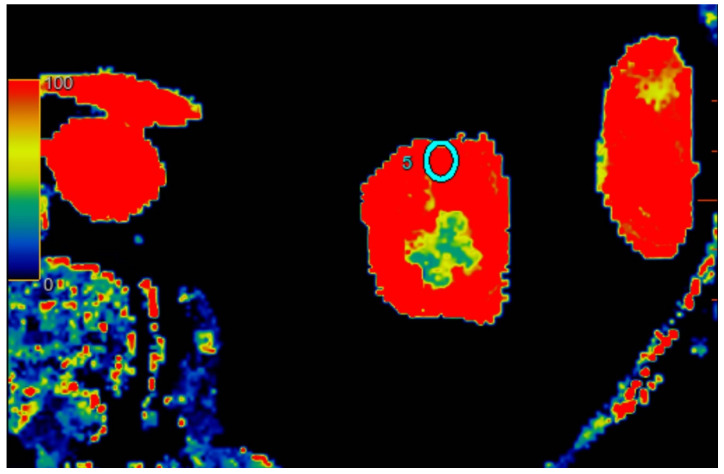
Visual representation of perfusion in CE-MDCT scans in the upper kidney pole with marked ROI (cyan oval). Cortical blood flow = 153.7 ± 52.9 mL/100 g/min; ROI area = 33.0 mm^2^.

**Figure 2:**
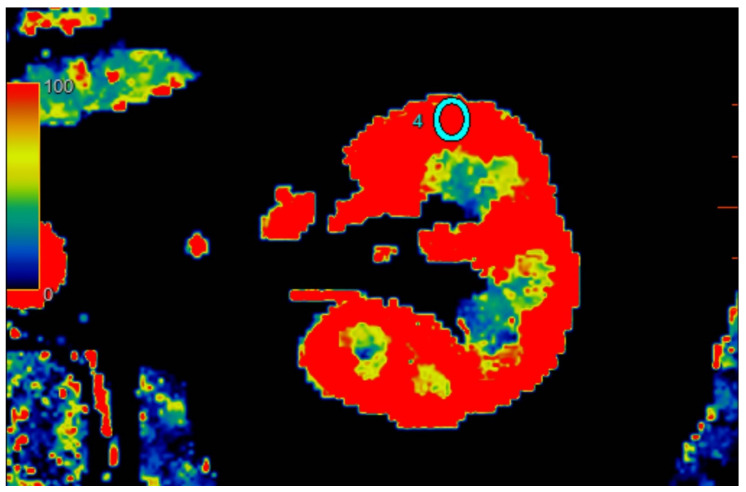
Visual representation of perfusion in CE-MDCT scans in the middle kidney pole with marked ROI (cyan oval). Cortical blood flow = 177.9 ± 43.9 mL/100 g/min; ROI area = 35.9 mm^2^.

**Figure 3:**
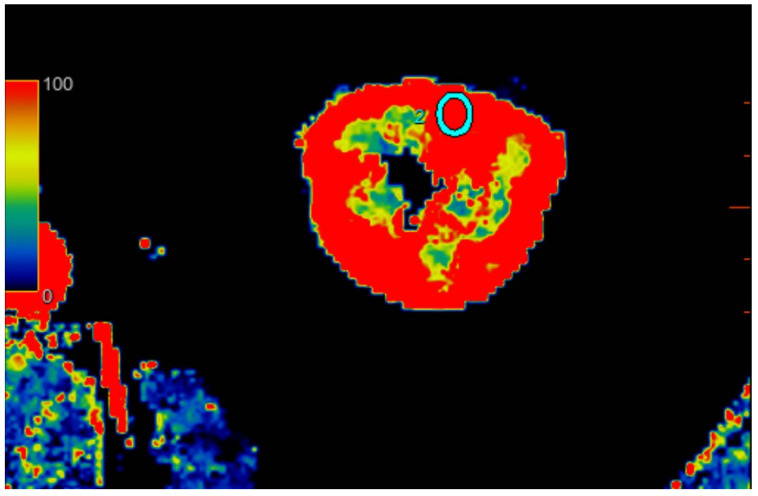
Visual representation of perfusion in CE-MDCT scans in the lower kidney pole with marked ROI (cyan oval). Cortical blood flow = 156.9 ± 43.9 mL/100 g/min; ROI area = 36.6 mm^2^.

All scans were examined with Advantage Workstation server 4.7 (GE Healthcare, USA), with software CT perfusion 4D. Scans were evaluated by one attending physician with 20 years of experience with computer tomography. The software automatically calculated the blood flow (mL/100 g/min) corresponding to ROI perfusion. Three calculations were performed for each kidney in different regions: upper kidney pole, middle of kidney, and lower pole. To examine regional perfusion, we used manually set ROIs, not smaller than 7 mm^2^, which were located in the kidney cortex, where artifacts were smallest and the examined area was the most homogenous during the observation cycle. All set ROIs were automatically transformed into a perfusion map with the software (Figure 1, Figure 2 and Figure 3).

### 2.3. Kidney Ultrasound

**Figure 4:**
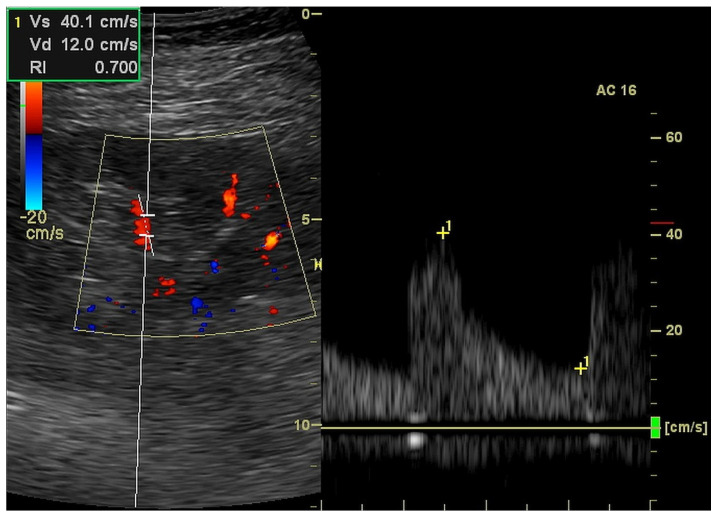
Ultrasound measurement of resistive index in the triplex (2D, color Doppler and pulse wave Doppler) mode.

We performed a kidney ultrasound examination (Logiq P6, GE Healthcare, Seoul, Korea; equipped with a curved array probe of 2–5 MHz) to measure the kidney length and to estimate the parameters of kidney perfusion. Two or three segmental arteries localized in different regions of each kidney were evaluated in color Doppler and pulsed-waived Doppler technics to measure acceleration (ACC (cm/s^2^)), acceleration time (ACC (ms)), resistive index (RI (ratio)) and end-diastolic velocity (EDV (cm/s)) based on Doppler wave spectrum analysis (Figure 4) [2].

**Figure 5:**
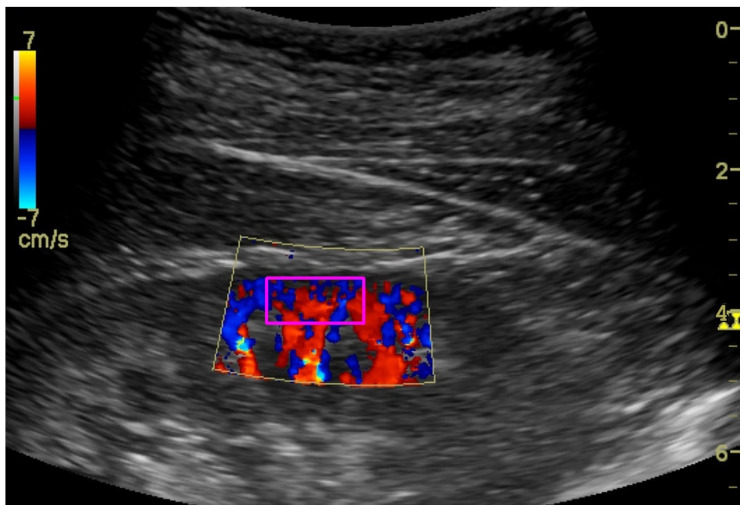
Ultrasound color Doppler visualization of renal cortex perfusion with marked (magenta rectangle) ROI.

To assess the renal cortex perfusion (RCP) parameters, the dynamic tissue perfusion measurement (DTPM) method was used [16,17]. In this technique, we set the gain of the color Doppler on a constant level to record comparable results. After identification of the middle cortical segment of the kidney (localized between two medullar pyramids) in the longitudinal projection, a color Doppler frame was activated between the pyramids and renal capsule (Figure 5). Then, 3–5 s clips were recorded and transferred to PC software (PixelFlux, Chameleon Software, Leipzig, Germany). Semi-automatic color Doppler clips analysis resulted in perfusion parameters: dynamic renal cortex perfusion intensity (dRCP (cm/s)), dynamic resistive index (dRI (ratio)), and the dynamic pulsatility ndex (dPI (ratio)), which were considered for statistical analysis.

## 3. Results

Thirty non-stenotic kidneys in twenty-five hypertensive patients (11M, 14F, age 58.9 ± 19.0) were included in the study. Twenty remaining kidneys met exclusion criteria because of nephrectomy (1 kidney) and >30% renal artery stenosis (19 kidneys). Demographic data and renal function of included patients are presented in Table 2. Three patients had CKD stage G1, seven had CKD G2, eleven had CKD G3, and four had CKD 4. Parameters of renal cortex perfusion estimated in CE-MDCT, conventional Doppler sonography, and DTPM are shown in Table 3.

### 3.1. Differences in CE-MDCT Measurements

**Figure 6:**
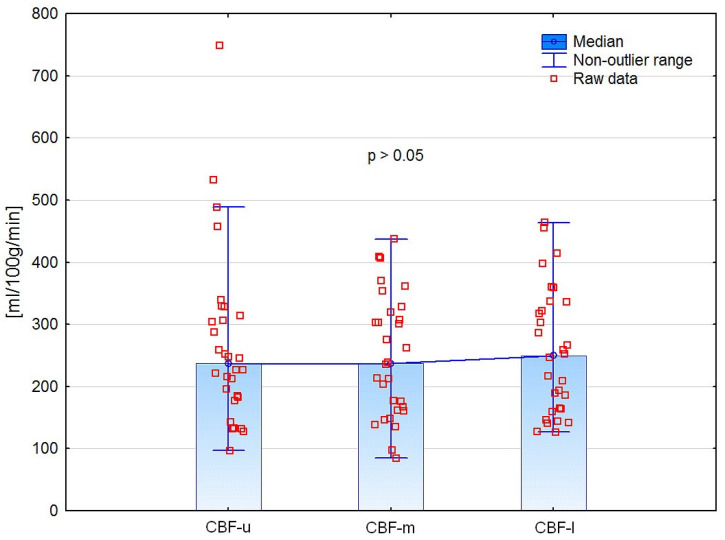
Comparison of CE-MDCT cortical blood flow in different kidney poles.

CE-MDCT results of renal cortical perfusion measured in different regions did not differ significantly (Figure 6). Moreover, we did not find any significant differences in perfusion parameters between the left and right kidneys (Table 4).

In opposite to the other CE-MDCT measurements, due to having the lowest standard deviation and close to normal distribution, the result of the CBF measurement in the middle kidney pole was set as a reference for further analyses. Moreover, this localization of CBF measurement was close to the DTPM region of interest, which reduced discrepancies between these two methods.

### 3.2. Associations of Renal Cortex Perfusion Parameters

CBF correlated significantly with age and eGFR. Moreover, RI, EDV, dRI, dPI, and dRCP were markedly related to CBF (Table 5).

In the model of stepwise backward regression analysis, including age, BMI, creatinine, and eGFR, only age independently influenced CBF (R^2^ = 0.317, *p* = 0.001). Further regression analyses in conventional Doppler parameters and the DTPM method showed that RI and dRCP were independently associated with CBF (R^2^ = 0.290, *p* = 0.003, and R^2^ = 0.320, *p* = 0.001, respectively) (Table 6). Lastly, from considered ultrasound parameters, stepwise backward regression analysis showed dRCP as the only variable independently related to CBF (R^2^ = 0.317, *p* = 0.001).

When the common ultrasound Doppler model was tested in stepwise forward regression analysis, only dRCP (as the first variable) and RI were independently related to CBF (R^2^ = 0.37, *p* = 0.003).

### 3.3. Relations of Perfusion Parameters with Kidney Function

Ultrasound flow parameters correlated with CBF were also associated with kidney function. RI (r = −0.459; *p* = 0.012), EDV (r = 0.672; *p* < 0.001), dRI (r = −0.534; *p* = 0.002), and dRCP (r = 0.707; *p* < 0.001) were significantly associated with eGFR. In the model of stepwise backward regression analysis concerning CBF, RI, EDV, dRI, and dRCP only CBF and EDV were independently associated with eGFR (R^2^ = 0.510, *p* < 0.001).

## 4. Discussion

In the presented study, we proved that renal cortex perfusion measured using the dynamic Color Doppler option and quantified in an external medical device (dRCP) is independently associated with renal cortical blood flow estimated in an objective CE-MDCT method (CBF). In recent years, the assessment of renal cortical perfusion has an increasing significance. Ma et al. investigated retrospectively 93 patients diagnosed for renal artery stenosis and found that RCP assessed in CEUS is correlated with renal function and the degree of stenosis [18]. In another work, renal cortex perfusion was the independent factor for renal function decline in 1 year of observation [19]. In the study conducted by Huo et al., semiquantitative estimation of renal blood flow was a good indicator of systemic and renal perfusion response to fluid resuscitation in patients with severe sepsis [20]. Although the DTPM method for renal cortex perfusion quantification was introduced over 15 years ago and is successfully used in many clinical applications, e.g., hypertension, glomerulonephritis, diabetic nephropathy, cardio-renal syndrome, vesicoureteral reflux, and renal neoplasm, it was not compared with a more objective and operator-independent method as CE-MDCT or magnetic resonance angiography [16,17,21,22,23,24,25].

In our work, considering all selected ultrasound flow parameters, dRCP estimated in the DTPM method had the strongest correlation with CBF. Nevertheless, from conventional ultrasound Doppler parameters, RI independently correlated with CBF. Thus, we confirmed that the renal resistive index measured in segmental renal arteries and the renal cortex perfusion estimated in the DTPM method are independently associated with the cortical blood flow in patients with hypertension and different stages of renal dysfunction. Recently, in the group of patients after thyroidectomy, we showed good repeatability of the DTPM method in estimating renal cortical perfusion [4]. Moreover, the diagnostic properties of RI were confirmed in earlier studies [1,14]. These data contribute to the use of the dynamic ultrasound assessment of renal cortex perfusion intensity (dRCP) or renal resistive index as perfusion markers in patients with hypertension and kidney disease. However, in hypotension, dehydration, sepsis, and shock, dRCP could be more accurate to CE-MDCT renal cortical blood flow than the RI [13,26]. Investigating acute kidney injury in a group of 50 patients with septic shock, Watchorn et al. showed that RCP measured in CEUS is independent of renal blood flow and RI [13]. However, this discrepancy can relate to 20% lower renal cortex perfusion than renal blood flow in a healthy state and derangement between renal macro- and microcirculation in septic patients [26]. On the other hand, the superiority of dRCP over RI in expressing CBF can be related firstly to the exact cortical ROI localization and secondly to more accurate flow properties in bigger ROI than a single vessel flow assessment [27,28]. Our findings firstly entitled the use of renal resistive index as a marker of kidney perfusion and secondly show the DTPM as a better ultrasound non-contrast method to estimate this feature. Although performing DTPM is time-consuming and somewhat complicated, by the use of external software, the RI measurement is widely used. Renal resistive index is recognized as a marker of vascular alterations in cardiovascular diseases [1]. It could be used, among others, for hypertension monitoring and cardiovascular risk estimation, acute kidney injury recovery prediction, chronic kidney disease diagnosing and prognosis, renal autoregulation and microvascular diabetic complications assessment, and renovascular hypertension diagnosis and treatment prognosis [1,3]. Investigating 5950 patients with hypertension, Radermacher et al. found 138 participants with renal artery stenosis > 50% [29]. They showed that a renal Resistive Index > 0.80 was associated with the lack of hypertension and renal function improvement after revascularization. Moreover, in another study, RI > 0.80 was connected with worse kidney outcomes and mortality despite the presence or absence of proteinuria [30]. Nevertheless, the elevated risk of unfavorable renal outcomes after revascularization probably starts with even lower RI values. Based on the case series, Cianci et al. suggested that RI > 0.75 at baseline and the absence of NGAL reduction after percutaneous renal artery angioplasty were associated with worsening renal function [31]. By showing an independent negative association of renal RI with kidney perfusion, we support a clear explanation for declining renal function in elevated RI circumstances.

For an objective assessment of renal perfusion, we used the low-dose (40 mL) CE-MDCT method as a reference to the ultrasound flow parameters. Using low-dose contrast agents could be associated with worse image quality and misdiagnosis. On the other hand, low-dose contrast-enhanced CT for perfusion assessment is recommended for all patients if the method is sufficiently diagnostic [32]. Similarly to our study, Asayama et al. used a low-dose contrast agent (40 mL) CT and compared image noise, overall quality, and perfusion parameters in reconstructed data of renal tumors with conventional contrast-enhanced CT [33]. Although image noise was higher and overall image quality was lower in low-dose CT than in conventional procedures, arterial visualization was improved, and perfusion parameters and tumor detection were comparable between these two techniques.

We investigated renal cortical blood flow in the CE-MDCT method in three different kidney localizations and found no significant differences in perfusion parameters. These data are consistent with the findings of Liu et al., who investigated renal perfusion in 43 patients with aortic dissection in 320-row CT using ROIs selected in four points at the axial or three points at the coronal plane (upper pole, hilum, and lower pole) of the kidney and found similarity of readings taken from these different positions [34].

Impaired renal perfusion can be the cause and the marker of kidney dysfunction. Although our results showed significant associations of Doppler parameters with cortical blood flow and kidney function, these relations were not obvious. The result of the dynamic assessment of renal cortex perfusion intensity was independently correlated with CE-MDCT cortical blood flow. Nevertheless, this association with kidney function was not as strong as CBF and EDV. Presented data are consistent with recent studies reporting a close relationship between EDV and kidney function, in contrast with other Doppler perfusion parameters that derive from the end-diastolic velocity [10,35,36].

Although our results are satisfactory, the presented study has some limitations. Firstly, the group is relatively small and encompasses patients with hypertension and mild-to-severe chronic renal impairment. Moreover, we investigated perfusion in nonstenotic kidneys in the population with hypertension diagnosed for renal artery stenosis. Since in contralateral to stenotic kidneys, renal vein inflammatory markers are significantly elevated, and inflammation could alter renal cortical perfusion, estimated values of perfusion parameters cannot be strictly related to healthy kidneys [37].

## 5. Conclusions

The color Doppler dynamic renal cortex perfusion intensity and renal resistive index are significantly associated with renal cortical perfusion in patients with hypertension and different stages of kidney dysfunction. Although the value of the Doppler dynamic tissue perfusion measurement method is superior in the renal cortex perfusion estimation, in the case of its inaccessibility, the conventional Doppler renal resistive index can be used to measure renal perfusion.

## Figures and Tables

**Table 1:** Technical data of CE-MDCT perfusion assessment protocol.

Phase	Tube Voltage (keV)	Tube Current (mAs)	Detector Coverage (mm)	Helical Thickness (mm)	Pitch/Speed (mm/rot)	Rotation Time (s)	Total Exposure Time (s)	DLP (mGy-cm)
I (non contrast)	120	170	40	5	1.375:1/55	0.6	2.12	105.94
Contrast agent: 40 mL (flow 4 mL/s) + bolus 20 mL 0.9% NaCl								
II (contrast)	100	(50–200)	40	5	1.375:1/55	0.4	42.6	Projected series −8.62 mGy-cm

DLP—dose length product.

**Table 2:** Demographic and kidney function characteristics of the investigated population.

	Mean	SD	Median	IQR
Age (years)	57.6	19.5	63.5	33.2
BMI (kg/m^2^)	26.5	4.2	26.3	6.5
Kidney length (mm)	107.4	12.4	108.4	13.0
Creatinine (mg/dL)	1.46	0.82	1.30	0.6
eGFR (mL/min/1.73 m^2^)	58.3	28.1	57.6	34.8

**Table 3:** Renal cortex perfusion parameters estimated in different methods.

	Mean	SD	Median	IQR
CBF-u	268.3	139.4	236.7	131.6
CBF-m	247.9	98.6	237.1	157.8
CBF-l	256.4	102.0	249.7	171.9
ACC (m/s^2^)	7.22	2.58	6.90	2.90
ACT (ms)	36.2	8.22	37.9	11.8
RI (ratio)	0.701	0.115	0.721	0.169
EDV (cm/s)	13.6	5.6	12.2	9.7
dRI (ratio)	0.735	0.168	0.760	0.205
dPI (ratio)	1.433	0.582	1.430	0.930
dRCP (cm/s)	0.483	0.452	0.303	0.529

ACC—acceleration; ACT—acceleration time; CBF—cortical blood flow in CE-MDCT; d—ultrasound dynamic tissue perfusion measurement; EDV—end-diastolic velocity; m—middle kidney pole; l—lower kidney pole; PI—pulsatility index; u—upper kidney pole; RCP—renal cortical perfusion; RI—resistive index.

**Table 4:** Differences between left and right kidneys.

	Left (*n* = 13)	Right (*n* = 17)	Significance—*p*
Kidney length (mm)	108.8 ± 14.3	106.4 ± 11.1	0.341
CBF-u (mL/100 g/min)	265.3 ± 127.5	270.7 ± 150.4	0.934 *
CBF-m (mL/100 g/min)	239.4 ± 99.5	254.4 ± 100.4	0.993
CBF-l (mL/100 g/min)	231.9 ± 94.5	275.2 ± 106.2	0.277 *
ACC (m/s^2^)	6.78 ± 1.65	7.56 ± 3.12	0.773 *
ACT (ms)	36.8 ± 8.8	35.7 ± 8.0	0.726
RI (ratio)	0.694 ± 0.121	0.707 ± 0.113	0.760
EDV (cm/s)	14.0 ± 5.3	13.2 ± 6.0	0.666
dRI (ratio)	0.741 ± 0.180	0.730 ± 0.165	0.730
dPI (ratio)	1.470 ± 0.592	1.410 ± 0.590	0.770
dRCP (cm/s)	0.539 ± 0.537	0.439 ± 0.387	0.680 *

*—Mann–Whitney U test.

**Table 5:** Analysis of correlations between CBF and other variables.

	Correlation Coefficient (*n* = 30)	Significance—*p*
Age (years)	−0.541 *	0.002
BMI (kg/m^2^)	−0.108	0.584
Kidney length (mm)	0.240	0.218
Creatinine (mg/dL)	−0.349 *	0.058
eGFR (mL/min/1.73 m^2^)	0.606	0.001
ACC (m/s^2^)	0.104 *	0.583
ACT (ms)	0.358	0.061
RI (ratio)	−0.513	0.005
EDV (cm/s)	0.444	0.018
dVmean (cm/s)	0.515	0.004
dRI (ratio)	−0.532	0.004
dRCP (cm/s)	0.581 *	<0.001

*—Spearman correlation coefficient.

**Table 6:** Results of the stepwise backward regression analyses.

	Demographic and Kidney Function Model	Conventional Ultrasound Doppler Model	Dynamic Tissue Perfusion Measurement Model	Common Ultrasound Doppler Model
Variables	Age	ACC	dVmean	RI
	Creatinine	ACT	dRI	EDV
	eGFR	RI	dRCP	dRI
	BMI	EDV		dRCP
Result:	Age	RI	dRCP	dRCP
R^2^	0.317	0.290	0.320	0.317
Significance—*p*	0.001	0.003	0.001	0.001

## Data Availability

The dataset is with the authors and available in scientific interest on request.
